# Mode of delivery and gut microbiota

**DOI:** 10.1186/1824-7288-40-S2-A17

**Published:** 2014-10-09

**Authors:** Vito Leonardo Miniello, Angela Colasanto, Lucia Diaferio, Laura Ficele, Maria Serena Leggi, Valentina Santoiemma

**Affiliations:** 1 Department of Paediatrics, Aldo Moro University of Bari, Bari, Italy

## 

In 1985 the World Health Organization (WHO) stated: "There is no justification for any region to have Caesarean Section (CS) rates higher than 10-15%"[1]. During the last decades the percentage of births managed by CS has increased beyond the recommended level, especially in high income areas such as Italy, Germany, France, United Kingdom, and North America [2,3].

Emerging evidences indicate that the early composition of neonatal gut microbiota is responsible for shaping of immune response since there is a complex interaction between the intestinal microbiome and the immune system (Gut-Associated Lymphoid Tissue) and this cross-talk is involved in maintaining normal immune homeostasis [4]. The microbiome promotes human health, but can also drive disease. The potential disadvantages of caesarean delivery include altered bacterial profile known as *dysbiosis* of the gut microbiota which in turn leads to immune dysfunction and increased tendency for immune-mediated diseases such as allergies [5,6] and autoimmunity [7].

Upon delivery, the neonate is exposed to a wide variety of microbes, many of which are provided by the mother during and after the passage through the birth canal, a heavily colonized ecosystem. The neonatal colonization pattern is further influenced by several post-natal environmental factors such as the place and mode of delivery, the level of affluence, the number of siblings, the use of antibiotics and infant feeding.

The reduced microbial exposure and delayed colonization occurring in caesarean born infants have been associated with the development of allergic disease. CS delivered infants, deprived of contact with the maternal vaginal microbiota, experience a deficiency of strict anaerobes such as *Bacteroides*, *E. coli*, and bifidobacteria and a higher presence of facultative anaerobes such as *Clostridium* species, compared with vaginally born infants [8].

It is debated whether a low total diversity of the gut microbiota during infancy is more important than an altered prevalence of particular bacterial species (*Clostridia*) for the increasing incidence of allergic disease [5,6]. Recently Bisgaard et al. demonstrated that reduced diversity of intestinal microbiota during infancy is associated with increased risk of allergic disease during childhood [9].

The concept of probiotics has attracted increasing attention in recent years since several clinical studies have been published suggesting that probiotics may convert a dysbiosis to a symbiosis in infants with inadequate intestinal colonization (premature delivery, delivery by CS and excessive use of perinatal antibiotics) [10-15]. Clinical evidences suggest that probiotics could substantially affect metabolic and immunomodulatory functions [16].
